# Case Report: The double-edged sword: cytokine release syndrome and rapid tumor response in a patient treated with amivantamab and chemotherapy

**DOI:** 10.3389/fonc.2025.1758359

**Published:** 2026-01-19

**Authors:** Pengfei Cui, Guannan Sun, Gang Zhou, Xueguang Guo, Haili Huang, Jing Yang, Na Li, Jing Tan, Yi Hu, Dong Zhang

**Affiliations:** 1 Department of Oncology, The Second Medical Center of Chinese People's Liberation Army General Hospital, Beijing, China; 2 Department of Neurosurgery, The 983rd Hospital of the People's Liberation Army, Tianjin, China; 3 Senior Department of Oncology, The Fifth Medical Center of Chinese People's Liberation Army General Hospital, Beijing, China

**Keywords:** amivantamab, case report, cytokine release syndrome, EGFR, non-small cell lung cancer

## Abstract

**Objective:**

To report a well-documented case of cytokine release syndrome (CRS) following the first cycle of amivantamab combined with chemotherapy in a patient with osimertinib-resistant EGFR-mutant non-small cell lung cancer (NSCLC), highlighting its diagnosis, management, and association with a rapid antitumor response.

**Case summary:**

A 72-year-old man with metastatic lung adenocarcinoma (epidermal growth factor receptor (EGFR) exon 19 del) progressing on osimertinib initiated treatment with amivantamab, pemetrexed, and carboplatin. Five days post-infusion, he developed a persistent fever unresponsive to broad-spectrum antibiotics. Infection was rigorously excluded based on normal procalcitonin, undetectable endotoxin, and negative cultures. In contrast, inflammatory markers were elevated (peak CRP (C−reactive protein) 16.6 mg/dL), and cytokine profiling revealed significant elevations in IL−6 (interleukin−6) (107 pg/mL), sIL-2R (soluble interleukin−2 receptor), TNF-α (tumor necrosis factor−alpha), and IL-10, accompanied by profound lymphopenia. A computed tomography (CT) scan demonstrated a significant tumor reduction. A diagnosis of grade 1 CRS (by ASTCT (American Society for Transplantation and Cellular Therapy) criteria) was made. The fever and inflammatory markers resolved with supportive care.

**Findings:**

This case provides comprehensive clinical and serological evidence of amivantamab-chemotherapy-induced CRS. The temporal association between the robust cytokine surge, characteristic of T-cell activation, and the observed rapid tumor regression suggests that the same immunologic mechanism likely underpinned both the efficacy and the toxicity.

**Conclusion:**

CRS is an important adverse event associated with amivantamab-based combination therapy in solid tumors. Early recognition, based on clinical suspicion and characteristic cytokine profiles, and differentiation from infection are crucial. The concurrent rapid tumor response indicates that effective immune activation drives both toxicity and efficacy, underscoring the need for optimal management strategies to mitigate CRS without compromising antitumor activity.

## Introduction

The treatment landscape for epidermal growth factor receptor (EGFR)-mutated non-small cell lung cancer (NSCLC) has been revolutionized by tyrosine kinase inhibitors (TKIs). Osimertinib, a third-generation TKI, is a standard of care. However, acquired resistance remains a major challenge, leading to limited options and poor outcomes (1).

Amivantamab is an epidermal growth factor receptor (EGFR) and mesenchymal–epithelial transition factor (MET) bispecific antibody with inherent immune cell-recruiting activity (2–4), has shown significant efficacy in clinical studies (5). It is now approved by the U.S. Food and Drug Administration (FDA) for use with chemotherapy in the second-line setting for patients with EGFR exon 19 deletions or L858R mutations whose disease progressed on prior EGFR TKI therapy (6, 7). Amivantamab exerts its effects through multiple mechanisms, encompassing ligand blockade, receptor degradation, and the engagement of effector cells (e.g., natural killer cells, monocytes, macrophages) via its optimized Fc domain (2, 3).

Cytokine release syndrome (CRS) is a potentially life-threatening systemic inflammatory response, characterized by a spectrum of worsening symptoms (e.g., fever, hypotension, hypoxia) and triggered by excessive immune activation involving T cells, B cells, NK cells, and macrophages, driven by a surge in pro-inflammatory cytokines such as interleukin-6 (IL-6) (8). Although CRS is well-documented with bispecific T cell engagers and chimeric antigen receptor T cell therapy (9, 10), it has also been increasingly reported with immune checkpoint inhibitors, particularly in combination regimens (11). For instance, a recent phase 3 trial (JCOG2007) investigating nivolumab plus ipilimumab combined with platinum-based chemotherapy in advanced NSCLC reported CRS in 3.4% of patients, with three fatal cases, highlighting the potential severity and unpredictability of CRS even outside classical T-cell engager therapies (12). As Synnott et al. (2025) highlight in their recent review, with the growing application of T-cell-engaging therapies like bispecific T-cell engagers (BiTEs) in solid tumors, there is a critical need to address CRS in this context, which may differ from hematological malignancies due to factors like the immunosuppressive tumor microenvironment (TME) (11). Although amivantamab-associated events (including a high incidence of infusion-related reactions) are well-documented in the pivotal trials that led to its approval (5), well-documented CRS cases with serial cytokine profiling, especially under amivantamab-chemotherapy combinations, remain scarce.

We report the first well-documented case, to our knowledge, of a patient with osimertinib-resistant EGFR exon 19 del NSCLC who developed definitive CRS after his first cycle of amivantamab plus chemotherapy, accompanied by a rapid tumor response. The novelty of this report lies in providing comprehensive clinical documentation alongside serial cytokine profiling, which supports the clinical diagnosis of CRS and distinguishes it from typical infusion-related reactions. This case aims to enhance clinical recognition of this entity and contribute to the understanding of its presentation and management in solid tumors.

## Case presentation

His oncological history began in July 2018 with a video-assisted thoracoscopic right upper lobectomy and lymph node dissection for stage pT1aN0M0 invasive adenocarcinoma with clear margins. The disease recurred in January 2020 with a local recurrence near the right pulmonary hilum and multiple bone metastases. An EGFR exon 19 deletion was identified through both blood-based and surgical specimen biopsies. He received first-line oral icotinib (125 mg three times daily) starting on February 4, 2020, alongside palliative radiotherapy (pGTV 45 Gy/10 fractions) to the thoracic spine metastases from May 27 to June 9, 2020, and denosumab (e.g., 120 mg subcutaneously every 4 weeks) for bone protection. A partial response was achieved, assessed according to the Response Evaluation Criteria in Solid Tumors version 1.1 (RECIST 1.1) (13). Upon progression in 2023, he was switched to Osimertinib (80 mg orally once daily). In May 2025, a new left upper lung nodule was treated with microwave ablation; biopsy confirmed poorly differentiated adenocarcinoma with retained EGFR exon 19 del, TP53 mutation, and PD-L1 (programmed death−ligand 1) expression of 40% (TPS (tumor proportion score)). Despite continued Osimertinib, serial imaging confirmed disease progression, with the left upper lobe mass enlarging from 12 × 9 mm (May 7, 2025) to 34 × 27 mm (September 8, 2025), and further to 34 × 39 mm (October 23, 2025), accompanied by new mediastinal lymphadenopathy and pleural effusion, confirming osimertinib resistance.

**Figure 1:**
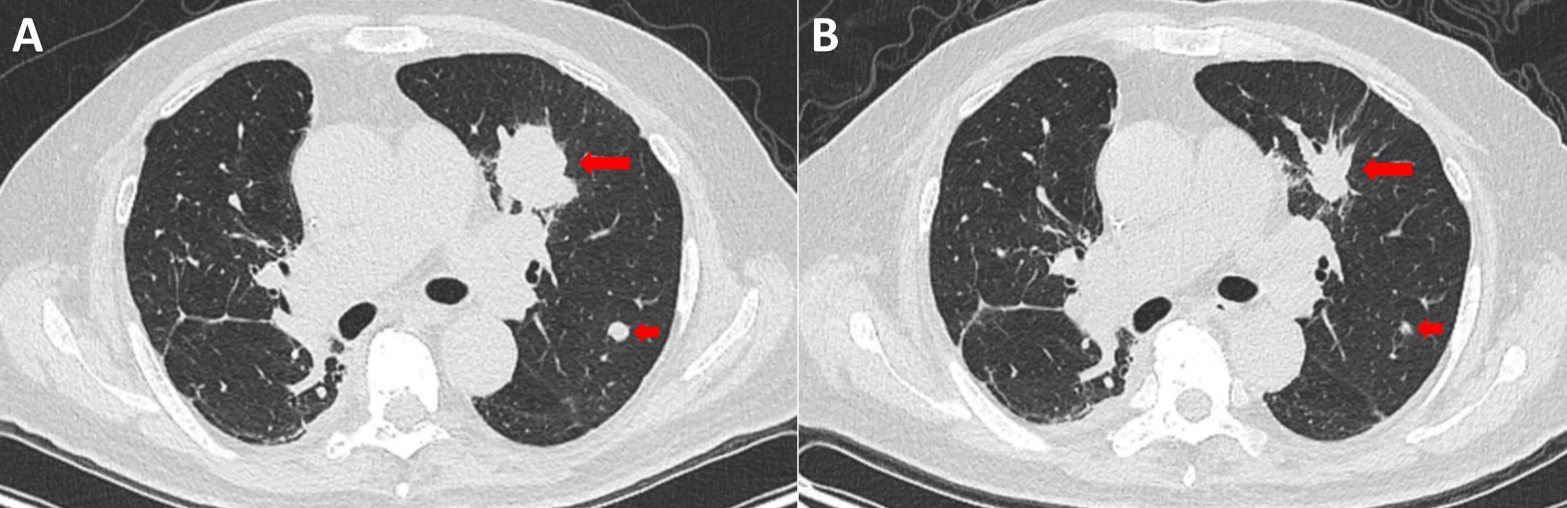
Contrast-enhanced chest CT images before and after treatment initiation. **(A)** Pre-treatment scan (October 23, 2025) shows multiple tumor lesions (red arrow) in the left upper lobe. **(B)** Scan obtained during the CRS episode following amivantamab-chemotherapy (November 5, 2025) demonstrates marked reduction in the size of the multiple lesions (red arrow).

On October 27, 2025, he started treatment with intravenous (IV) amivantamab (350 mg on day 1, 1050 mg on day 2), pemetrexed (800 mg, single dose, IV), and carboplatin (300 mg, single dose, IV). Starting November 1, he developed a 10-day persistent fever (38.2°C–38.9°C) with sinus tachycardia (105–115 bpm). Notably, he did not present with respiratory (e.g., cough, sputum) or gastrointestinal (e.g., diarrhea) symptoms. However, small (approximately 5 mm in diameter), slightly elevated, non-pruritic, erythematous papules appeared on the anterior chest concurrently with the onset of fever and resolved as the fever subsided. His blood pressure remained stable (110–135/70–85 mmHg) without vasopressor requirement, and oxygen saturation was maintained >96% on room air. Liver and renal function tests were normal. Empirical broad-spectrum antibiotics were administered without clinical response: piperacillin-tazobactam (IV, from Nov 2), which was escalated to meropenem plus vancomycin (IV, from Nov 4 to Nov 7). The patient had been previously vaccinated against SARS-CoV-2 in 2021, and rapid antigen/PCR tests for SARS-CoV-2 and influenza A/B at fever onset were negative. Crucially, a follow-up CT scan on November 5, 2025, which showed no signs of pulmonary infection, revealed significant tumor reduction (an approximate 63.5% reduction in tumor volume, calculated from 34 × 39 mm to 26 × 18 mm) compared to the October 23 scan (**Figure 1**).

**Figure 2:**
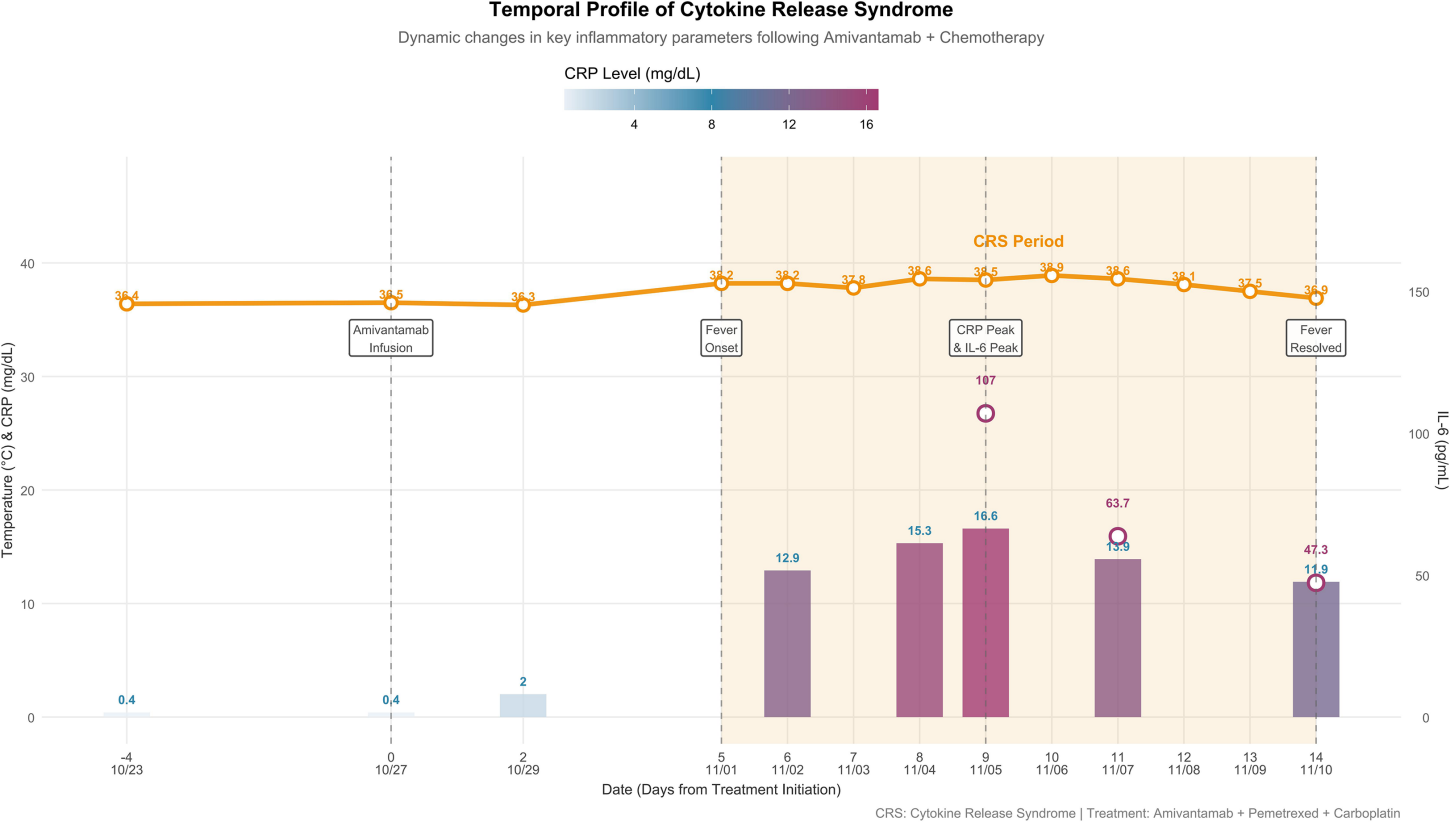
Temporal dynamics of key inflammatory parameters during cytokine release syndrome (CRS) following amivantamab combination therapy. The timeline illustrates the dynamic changes in body temperature (orange line and points, °C), C-reactive protein (CRP, bars, mg/dL), and interleukin-6 (IL-6, purple open circles, pg/mL) from baseline through CRS resolution. Key clinical events are annotated below the timeline: amivantamab infusion (Day 0), fever onset (Day 5), concurrent peak of CRP and IL-6 (Day 9), and fever resolution (Day 14). The shaded orange area highlights the CRS period, characterized by sustained fever, marked elevation of inflammatory markers, and rapid tumor response observed on Day 9. IL-6 levels were measured at three timepoints (Days 9, 11, and 14). Note: Cytokine measurement began on Day 9; the true peak may have occurred earlier.

Given the discordance between persistent fever and excellent tumor response, cytokine release syndrome (CRS) was suspected. A comprehensive laboratory workup was performed to rule out infection and characterize the inflammatory response. On November 4, 2025, whole blood samples were collected for endotoxin testing and cultures for aerobic/anaerobic bacteria and fungi, given the onset of fever. The complete blood count at this time showed an absolute neutrophil count within the normal range, arguing against febrile neutropenia. On November 5, 2025, key assessments were made. Procalcitonin was 0.22 ng/mL (normal range <0.5 ng/mL at our institution). In contrast, inflammatory markers were elevated: C-reactive protein (CRP) had risen to 16.6 mg/dL (from a pre-treatment baseline of <0.4 mg/dL on October 23). Cytokine profiling of plasma samples via electrochemiluminescence assay revealed a marked elevation of interleukin-6 (IL-6) at 107 pg/mL. A concurrent CT scan confirmed significant tumor reduction. A limitation is that cytokine measurements commenced four days after fever onset; therefore, the true cytokine peak may have been earlier and potentially higher than recorded. On November 7, 2025, follow-up evaluations showed a 40.5% decrease in IL-6 level to 63.7 pg/mL. Cytokine profiling additionally identified elevated levels of soluble interleukin-2 receptor (sIL-2R; 1000 U/mL), tumor necrosis factor-alpha (TNF-α; 23.60 pg/mL), and interleukin-10 (IL-10; 7.23 pg/mL), while interleukin-1 beta (IL-1β) was measured at 5.0 pg/mL, within the manufacturer-provided reference range for healthy individuals (0–5 pg/mL), all measured from plasma via electrochemiluminescence assay. Lymphocyte subset analysis by flow cytometry on peripheral blood samples revealed profound lymphopenia (total lymphocyte count 763/μL; reference range 1530–3700/μL), with reductions in CD4+ T cells (346/μL), CD8+ T cells (140/μL), and NK cells (46/μL). Procalcitonin remained normal (0.14 ng/mL), and the blood cultures from November 4 were ultimately reported as negative. By November 10, 2025, with supportive care, the IL-6 level had further declined to 47.3 pg/mL (a 55.8% reduction from the November 5 peak), and CRP trended down to 11.9 mg/dL. The dynamic changes of these parameters are summarized in **Figure 2**.

A diagnosis of grade 1 cytokine release syndrome (CRS) was made according to the American Society for Transplantation and Cellular Therapy (ASTCT) consensus criteria, which are based on clinical parameters (14). The patient met the criteria for grade 1 CRS due to the presence of fever (with a documented peak of 38.9 °C on November 6) without evidence of hypoxia (oxygen saturation maintained >96% on room air) or hypotension (blood pressure stable without vasopressor requirement). Supportive care, oral antipyretics (e.g., acetaminophen), was provided from November 1 to 10. No immunosuppressive agents were required. With this management, his fever gradually abated from November 8 and resolved by November 10, a course that coincided with the improvement of inflammatory markers, specifically the decline in interleukin-6 (IL-6) and CRP levels. The significant tumor shrinkage observed on November 5 was subsequently confirmed as a partial response according to RECIST 1.1 criteria.

Following resolution of the initial CRS episode, the patient proceeded with the planned treatment course. He subsequently received one cycle of amivantamab monotherapy followed by one cycle of amivantamab combined with pemetrexed and carboplatin. Both subsequent cycles were administered with prophylactic corticosteroids and closer monitoring. No recurrent CRS or significant infusion-related reactions occurred. The most recent follow−up imaging (performed after the third cycle of therapy overall) confirmed a sustained partial response (PR). While the tumor burden continued to regress compared to baseline, the degree of shrinkage was less pronounced than the dramatic response observed immediately after the first cycle. The patient remains on therapy with good tolerance and ongoing clinical benefit.

### Ethics approval and consent to participate

Ethical approval for this case report was waived by the Institutional Review Board of the Ethics Committee of the Chinese People’s Liberation Army General Hospital with number S2018-092-01. Written informed consent was obtained from the patient for publication of this case report and any accompanying images.

## Discussion

**Figure 3:**
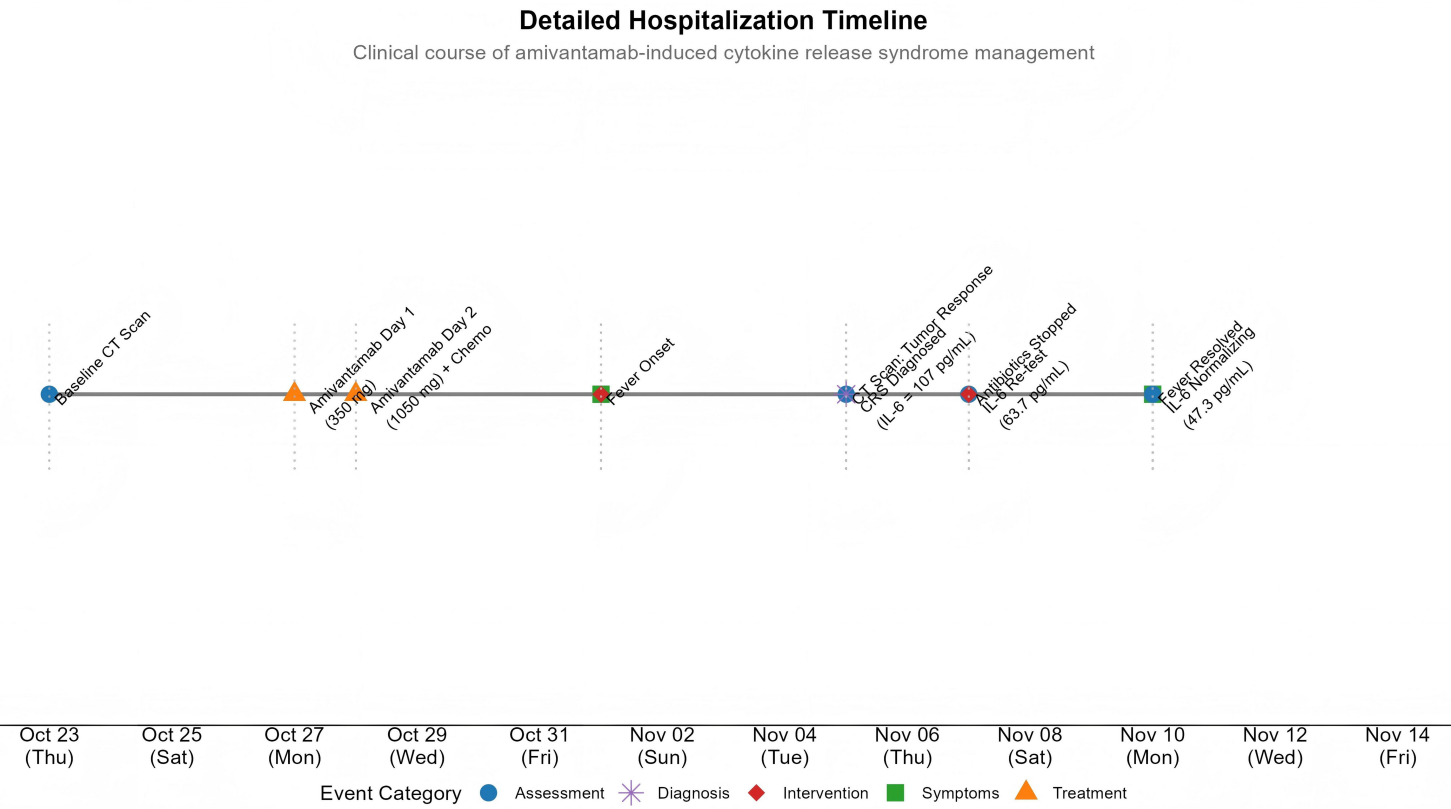
Clinical timeline of hospitalization for cytokine release syndrome management. The timeline illustrates the chronological sequence of key clinical events during the patient’s hospitalization. Events are categorized by type and marked with distinct colors and shapes: assessment (blue circles), treatment administration (orange triangles), symptoms (green squares), interventions (red diamonds), and diagnosis (purple stars). The progression from amivantamab infusion (Oct 27-28) through CRS development and resolution (Nov 1-10) is delineated.

To our knowledge, this report provides the first comprehensive account of characterized CRS induced by amivantamab in combination with chemotherapy, offering valuable insights aligned with and expanding upon recent scholarly discourse. Our report directly addresses the emerging challenge outlined by Synnott et al.—the characterization of CRS in solid tumors as novel immunotherapies like immune checkpoint inhibitors (ICIs) gain traction (11). The novelty of this case lies in its status as the first reported instance of diagnosed CRS with supporting cytokine data following amivantamab-chemotherapy combination. It is noteworthy that in the pivotal clinical trials leading to amivantamab’s approval—such as the CHRYSALIS (monotherapy) and MARIPOSA-2 (post-osimertinib with chemotherapy) studies—adverse events like fever were primarily categorized and managed as infusion-related reactions (IRRs), with detailed CRS characterization not reported (5). Our report provides robust serological confirmation with serial cytokine profiling (elevated IL-6, sIL-2R, TNF-α, IL-10) and rigorous exclusion of infection, fulfilling the ASTCT criteria for CRS (15). This distinction is crucial, as it highlights a specific immunotoxicity mechanism beyond typical IRR, linking robust systemic immune activation directly to both significant toxicity (CRS) and remarkable efficacy (rapid tumor regression). We acknowledge that in this combination regimen, the contribution of chemotherapy to the observed lymphopenia and overall clinical picture cannot be entirely excluded, although the cytokine profile and timing are most consistent with an amivantamab-driven immune activation. The overall timeline of key clinical events is depicted in **Figure 3**.

First, our case unequivocally establishes the diagnosis of CRS. According to the ASTCT consensus criteria (15), CRS is defined and graded based on clinical parameters (fever, hypotension, hypoxia), and the patient’s symptoms (fever onset 5 days post-infusion without respiratory/intestinal infection symptoms) met the criteria for grade 1 CRS. The biochemical evidence is robust: significant elevation of IL-6, a central pathogenic cytokine in CRS (11, 16), marked increase in sIL-2R (a direct marker of T-cell activation) (9), and elevated TNF-α. Notably, IL-1β was measured at the peak of inflammation (November 5) and was within the normal range (5.0 pg/mL). This cytokine profile—elevated IL-6, TNF-α, and IL-10 with normal IL-1β—exhibits a distinct pattern from syndromes primarily driven by the canonical IL-1β/IL-6 axis (14), suggesting a different immunologic etiology for the CRS in this case. The observed elevation of IL-6, sIL-2R, and TNF-α is consistent with the cytokine profile seen in CRS following T-cell activation. Notably, the IL-6/IL-6R axis is recognized as a pivotal driver in CRS associated with T-cell immunotherapies (10), and IL-6 is routinely monitored in the characterization of T-cell–engaging bispecific antibody (T-BiSp)-induced CRS (17). As emphasized in the seminal review by Fajgenbaum and June, while cytokine levels are not diagnostic criteria, they are central to understanding CRS pathophysiology (18). Our observation of elevated IL-6, TNF-α, and IL-10—cytokines strongly associated with activation of innate immune cells like macrophages—aligns with this paradigm. The parallel finding of profound lymphopenia further completes the pathophysiological picture, likely reflecting massive immune cell activation and consumption (9), though a contributory effect from concurrent carboplatin and pemetrexed chemotherapy is possible. This aligns with the pathophysiology described by Synnott et al. (11).

The initial presentation of persistent fever following potent immunotherapy necessitated a rigorous exclusion of infectious etiologies, a process in which the patient actively participated by reporting detailed symptom changes. Several lines of evidence collectively argued against infection as the cause. First, the clinical presentation was atypical for common infections: the patient lacked localizing symptoms such as cough, sputum production, or diarrhea, and specifically reported the novel appearance of a rash as his main concern alongside fever. Instead, the appearance of small, non-pruritic erythematous papules concurrent with the fever trajectory is more consistent with a systemic inflammatory state like CRS (16). Second, key investigative findings were negative: the diagnostic CT scan on November 5 served a dual purpose, confirming a tumor response while simultaneously showing no radiographic evidence of pneumonia. Furthermore, the normal level of IL-1β, a pyrogen typically elevated in certain severe bacterial infections and autoinflammatory syndromes, provided additional biochemical support against these specific infectious or inflammatory mimics. Rapid molecular and antigen testing for prevalent respiratory pathogens (SARS-CoV-2, influenza A/B) at fever onset was negative, and the patient had a history of SARS-CoV-2 vaccination. Critically, procalcitonin levels remained low, and blood cultures showed no growth. Third, and crucially, the fever exhibited no response to a sequential course of broad-spectrum antibiotics (piperacillin-tazobactam escalated to meropenem/vancomycin). The temporal coincidence of this refractory fever with a dramatic on-treatment tumor reduction creates a compelling link, suggesting that the systemic inflammatory syndrome and the antitumor immune response shared a common driver—the robust immune activation induced by amivantamab.

The most intriguing finding is the dramatic tumor regression observed during peak CRS. This temporal association strongly suggests that the same robust immune activation responsible for CRS was simultaneously driving effective anti-tumor immunity. This observation supports the paradigm that effective T cell activation underpins both efficacy and toxicity, and that managing CRS does not necessarily abrogate antitumor responses. This concept is supported by evidence from other T-cell engaging therapies, such as CAR-T cells, where the management of CRS is often successfully decoupled from the persistence of antitumor activity (9). This reinforces that effective toxicity management aims to modulate deleterious systemic inflammation without abolishing the anti-tumor response.

Furthermore, the patient’s high PD-L1 expression (TPS 40%) invites hypothesis-generating mechanistic speculation. A pre-existing immunologically active tumor microenvironment (TME), as suggested by high PD-L1, might have predisposed him to a more vigorous immune response. This is supported by literature indicating that EGFR signaling can modulate PD-L1 expression in the TME (19), potentially creating a context where amivantamab induces a stronger inflammatory and anti-tumor effect. However, we emphasize that this is speculative, as amivantamab does not directly target the PD-1/PD-L1 axis, and a direct causal link cannot be established from this single case. This resonates with discussions on how the solid TME can influence immune reactions (11).

Notably, the clinical course and management of CRS in our case differ from those reported with immune checkpoint inhibitor-based combinations. In the JCOG2007 trial, CRS associated with nivolumab plus ipilimumab and chemotherapy occurred at variable timepoints and carried a high mortality rate (12). In contrast, our patient, after being closely monitored and reassured, developed CRS shortly after the first infusion and recovered with supportive care alone, underscoring the spectrum of CRS severity and the importance of early recognition and tailored management. For severe CRS (typically grade ≥2 with organ dysfunction), escalation to immunosuppressive therapy, such as the IL-6 receptor antagonist tocilizumab, should be considered per established guidelines (20).

This study has inherent limitations as a single-case report, which restricts generalizability. The data were collected retrospectively. Although we measured IL-1β and found it to be normal, our cytokine panel was not exhaustive. Other unmeasured mediators (e.g., IL-18, IFN-γ) could provide additional mechanistic insights. The specific cellular subsets driving the response remain uncharacterized. Future prospective studies with systematic immune monitoring (e.g., single-cell RNA sequencing) are warranted to validate these findings and elucidate the precise mechanisms linking amivantamab, CRS, and efficacy. Importantly, incorporating standardized patient-reported outcome measures in such studies would better capture the patient experience of these adverse events.

In conclusion, this report alerts clinicians to CRS as a potentially serious adverse event under amivantamab-based combination therapy for solid tumors. It provides a framework for its diagnosis and differential diagnosis, highlighting that robust immune activation, while manifesting as toxicity, can be the very driver of profound anti-tumor efficacy. As emphasized by Synnott et al. (11), a deeper understanding of CRS in solid tumors will enable more personalized treatment approaches, enhancing the safety and efficacy of immunotherapies for this patient population.

## Data Availability

The original contributions presented in the study are included in the article/supplementary material. Further inquiries can be directed to the corresponding authors.
